# Raccoon spatial ecology in the rural southeastern United States

**DOI:** 10.1371/journal.pone.0293133

**Published:** 2023-11-09

**Authors:** Jacob E. Hill, Madison L. Miller, James L. Helton, Richard B. Chipman, Amy T. Gilbert, James C. Beasley, Guha Dharmarajan, Olin E. Rhodes

**Affiliations:** 1 Savannah River Ecology Laboratory, University of Georgia, Aiken, SC, United States of America; 2 Warnell School of Forestry and Natural Resources, University of Georgia, Athens, GA, United States of America; 3 National Rabies Management Program, USDA, APHIS, Wildlife Services, Concord, NH, United States of America; 4 National Wildlife Research Center, USDA, APHIS, Wildlife Services, Fort Collins, CO, United States of America; 5 Odum School of Ecology, University of Georgia, Athens, GA, United States of America; University of South Carolina, UNITED STATES

## Abstract

The movement ecology of raccoons varies widely across habitats with important implications for the management of zoonotic diseases such as rabies. However, the spatial ecology of raccoons remains poorly understood in many regions of the United States, particularly in the southeast. To better understand the spatial ecology of raccoons in the southeastern US, we investigated the role of sex, season, and habitat on monthly raccoon home range and core area sizes in three common rural habitats (bottomland hardwood, upland pine, and riparian forest) in South Carolina, USA. From 2018–2022, we obtained 264 monthly home ranges from 46 raccoons. Mean monthly 95% utilization distribution (UD) sizes ranged from 1.05 ± 0.48 km^2^ (breeding bottomland females) to 5.69 ± 3.37 km^2^ (fall riparian males) and mean monthly 60% UD sizes ranged from 0.25 ± 0.15 km^2^ (breeding bottomland females) to 1.59 ± 1.02 km^2^ (summer riparian males). Males maintained home range and core areas ~2–5 times larger than females in upland pine and riparian habitat throughout the year, whereas those of bottomland males were only larger than females during the breeding season. Home ranges and core areas of females did not vary across habitats, whereas male raccoons had home ranges and core areas ~2–3 times larger in upland pine and riparian compared to bottomland hardwood throughout much of the year. The home ranges of males in upland pine and riparian are among the largest recorded for raccoons in the United States. Such large and variable home ranges likely contribute to elevated risk of zoonotic disease spread by males in these habitats. These results can be used to inform disease mitigation strategies in the southeastern United States.

## Introduction

The spread of zoonotic diseases from wildlife to domestic animals and humans is of increasing concern to human health and may have significant economic impacts [1]. Several attributes of wildlife spatial ecology influence zoonotic disease prevalence and spread such as host home range sizes, migration and dispersal [2]. For many species, these parameters vary by habitat in accordance with resource availability, necessitating habitat-specific management strategies [2, 3]. Therefore, an understanding of wildlife spatial ecology across habitats is integral to the effective management of zoonotic diseases [2].

Across the United States, rabies virus poses a threat to the health of humans, domestic animals, and endangered species [4]. Raccoon (*Procyon lotor*) rabies virus (RABV) is the major rabies variant in the eastern United States, where raccoons are the primary reservoir for rabies virus [4]. The principal technique for landscape level wildlife RABV control in North America is preventive vaccination of wildlife via the coordinated deployment of baits containing an RABV vaccine, a strategy known as oral rabies vaccination (ORV). On average, 8–10 million ORV baits are distributed in the United States annually, about 90% of which are deployed aerially across rural landscapes using fixed-wing aircraft [4, 5]. By creating a vaccine border along the Appalachian Mountains, the United States Department of Agriculture’s National Rabies Management Program aims to prevent western spread of raccoon RABV and ultimately eliminate rabies from the eastern United States [6].

Raccoons (*Procyon lotor*) are generalist mesopredators that occupy diverse native habitats including pine forests, hardwood forests, prairies, and wetlands, in addition to anthropogenic habitats such as agricultural and urban areas [7–9]. Across this spectrum of habitats, raccoon home range sizes may vary widely in accordance with the abundance and availability of resources such as food, free water, and denning habitat [9–12]. In urban and agricultural habitats with high resource concentrations, raccoons need to travel less widely to meet resource requirements, resulting in smaller home ranges and greater population densities [7, 9, 10, 13–15]. Conversely, in habitats consisting of more natural land cover, raccoons tend to exhibit larger home ranges and lower population densities due to greater resource dispersion [9, 10, 13].

The movement behavior of raccoons influences patterns of pathogen transmission at the landscape level which in turn influences the effectiveness of ORV efforts [16]. Raccoons that display a high degree of variability in home range sizes over time are more likely to spread rabies due to increased rates of contact with conspecifics over long distances [17]. Such individuals may negatively affect the success of ORV efforts by breaching vaccination zones [17]. Additionally, models of raccoon RABV spread incorporate movement behavior by infected raccoons, which influences their likelihood of overlapping space use with conspecifics and spreading RABV [18, 19]. Habitat-specific patterns of raccoon space use can be employed to tailor ORV baiting strategies to certain habitats when desirable [16, 20]. These factors make a more comprehensive understanding of the drivers of wildlife movement behavior a priority for RABV management [16, 17, 21, 22].

Despite the relevance of raccoon home range ecology for ORV and disease management, many habitats are data deficient regarding patterns in raccoon home range sizes, including rural non-agricultural habitats of the Southeastern United States where raccoon space use may be influenced by many variables. Raccoons likely have smaller home ranges in resource abundant habitats such as bottomland hardwoods and riparian forests, which contain ample free water and large diameter trees for denning [23–25]. Upland pine forests, on the other hand, tend to have less suitable trees for denning, less water, and decreased availability of soft mast, an important food for raccoons in natural areas, likely leading to lower density raccoon populations with larger home ranges [8, 11, 23, 24]. Male raccoons usually have larger home ranges than females due to increased resource requirements and their attempts to overlap with the home ranges of multiple females to maximize mating opportunities [7, 24, 26]. Additionally, there may be seasonal changes in home range sizes as both sexes may range more widely during the breeding season due to mating activities [8, 26, 27]. In the summer, which overlaps with the period of weaning, home ranges may decrease owing to seasonal increases in food availability and limited movements of females during weaning activities [8, 27, 28].

In addition to home ranges, core areas are an important component of space use as they comprise areas of concentrated resources within the home range [29]. We used GPS collars to calculate monthly home ranges and core areas of raccoons in three rural habitats (bottomland hardwood, riparian, and upland pine) to test the hypothesis that space use of raccoons in southeastern rural habitats varies in size by sex, season and habitat. We predicted larger home ranges and core areas for animals in upland pine compared to bottomland hardwood and riparian, and for males compared to females. We also predicted home ranges and core areas would be largest during the breeding season and smallest during summer.

## Methods

### Study area

**Fig 1:**
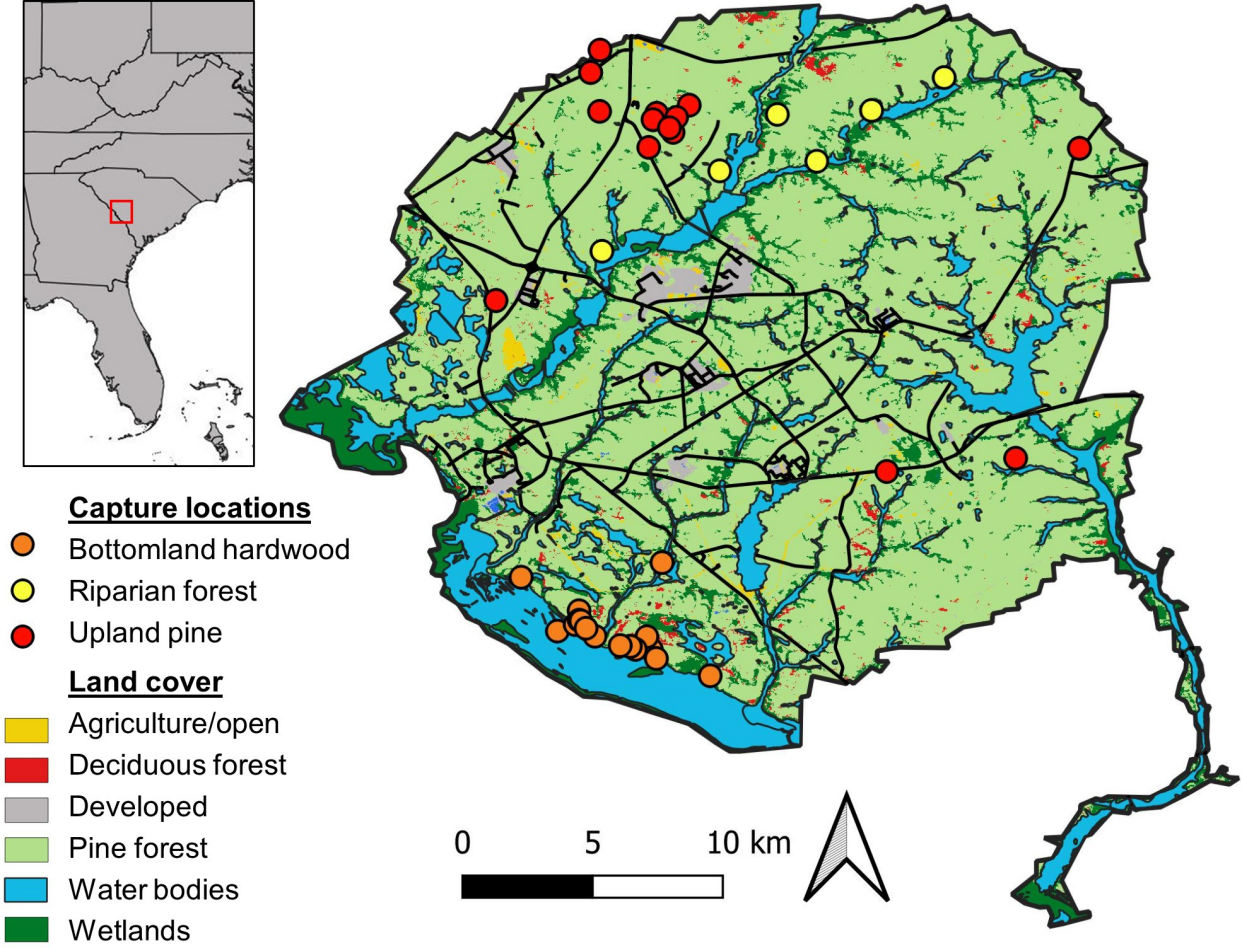
Map of the Savannah River Site, South Carolina, USA showing capture locations of raccoons by habitat. Maps were generated using Natural Earth (http://www.naturalearthdata.com) and the National Land Cover Database [31].

We conducted this study on the Savannah River Site (SRS), a 780 km^2^ site owned by the United States Department of Energy in the upper Coastal Plain region of South Carolina, USA (33°19’N, 81°42’W; Fig 1). The SRS was established in the 1950’s as a nuclear production facility and operations today consist of facilities for nuclear materials processing, tritium extraction and waste disposal [30]. Since 1951, much of the SRS has been managed for timber harvest (originally slash pine [*Pinus elliottii*] and subsequently loblolly [*Pinus taeda*] and longleaf pine [*Pinus palustris*]), and pine plantations are harvested on a rotating basis and subject to management practices such as thinning and prescribed burning [30]. Today the SRS is covered mostly by evergreen forest (54%) and woody wetlands (24%), with other land cover types (e.g., developed, open water, mixed forest) collectively comprising 22% of the land area [31]. Public access is restricted on the SRS and hunting of raccoons is not permitted. The nearest potentially hunted raccoon populations to the site exist on the Crackerneck State Wildlife Management Area (WMA) where racoon hunting is only allowed on a few nights in January and February. Crackerneck WMA was about 12 km away from our closest trapping grid and there is a minimal chance that harvest impacted movement or behavior of raccoons in our study.

We studied raccoon movement patterns in three prominent habitats on the SRS: upland pine, bottomland hardwoods and riparian. Upland pine is characterized by mature stands of loblolly and longleaf pine (*Pinus palustris*) with land cover classified as evergreen by the National Land Cover Database (NLCD). Bottomland hardwoods are classified as woody wetlands by the NLCD and are confined to the lower southwest portion of the site along the Savannah River and consist of seasonally flooded cypress-tupelo forests (*Taxodium distichum*-*Nyssa aquatica*), with oak (*Quercus* spp.) and hickory (*Carya* spp.) scattered throughout [30]. Riparian forest is also classified as woody wetlands, but bottomland hardwood is largely one contiguous habitat on the SRS. In contrast, riparian forests are more fragmented and are embedded in a matrix of upland habitat such as pine and hardwoods, existing in relatively narrow corridors along smaller rivers and creeks that feed into the Savannah River. This habitat is commonly produced by land conversion where native vegetation along waterways is left intact, resulting in the formation of a riparian zone [32]. Our riparian habitats were located along the upper portions of Tinker Creek and the Upper Three Runs Creek, both of which are relatively undisturbed and never received thermal effluent from nuclear reactors [33, 34].

### Field methods

Raccoons used for radio-tracking were captured as part of a concurrent study to evaluate raccoon abundance [35]. To trap raccoons, we established six trapping grids consisting of 25 Tomahawk^®^ model 108SS live-capture box traps (Hazelhurst, WI, USA) at intervals of 100 m in a 5 x 5 square configuration within each of the three focal habitat types (total of 18 trapping grids). Each grid was trapped for 2 sessions both consisting of 10 consecutive days, a spring (February-March 2021) and autumn (October-December 2021) session. Whole kernel corn was placed on the ground adjacent to the trap and plaster tabs soaked in fish oil were placed inside the traps as a lure [36]. We replaced the tabs after capture events and halfway through the trapping sessions. Corn was replaced as needed on daily checks of traps.

To reduce stress to the animal, we placed traps away from roads or other visible areas, covered them with vegetation, and approached trapped animals slowly and calmly. As raccoons are nocturnal, we checked traps immediately after sunrise so that animals remained in the traps for less than 12 hours. We anesthetized raccoons upon capture using intramuscular injection of Telazol (Fort Dodge Animal Health, Fort Dodge, IA) at a dosage of 5 mg/kg of estimated body weight [37–39]. We administered ophthalmic ointment to prevent drying of the eyes, and placed cloth masks over the face to calm the animal. Once anesthetized, we determined sex, age, and body mass and gave each raccoon a unique numbered ear tag (Monel #3, National Band and Tag Company, Newport, KY). To ensure the collar weight (115 g) was less than 5% of total animal weight, only animals that weighed 2.3 kg or more received transmitters [40, 41]. Following all handling procedures, we placed animals in an inconspicuous location out of direct sunlight near the capture site and observed them until full recovery.

We collected spatial data using GPS telemetry data logging transmitters (W500-AA, Advanced Telemetry Systems, Isanti, MN) programed to collect data points once every two hours between 18:00 and 6:00 hours, plus an additional point at 12:00. We chose this sampling schedule to maximize battery life; consistent daytime locations were of limited use because raccoons are mostly nocturnal and typically inactive in dens during the day. Each collar was outfitted with a very high frequency (VHF) transmitter that allowed raccoons to be located via radio telemetry for collar downloads. Data were remotely downloaded from each collar using an ultra-high frequency (UHF) antenna during daylight hours while raccoons were denning. We downloaded data at approximately monthly intervals until collar failure, loss of signal, or through July 2022.

We initially deployed 18 collars in spring 2021 divided evenly among sexes and habitats (3 per sex per habitat). By autumn 2021, some individuals had not been relocated for data downloading and thus we deployed an additional 6 collars in sex and habitat treatments that were data deficient. After deployment of these collars, we relocated and retrieved data from some of the missing individuals that had been collared in the spring, resulting in uneven sample sizes. Our final sample sizes (number of individuals monitored) were: 6M, 4F in bottomland hardwoods; 3M, 3F in upland pine; and 4M, 4F in riparian forest. To supplement our dataset, we also used telemetry data from an additional 22 raccoons trapped in the same bottomland hardwood (5M, 5F) and upland pine (6M, 6F) trapping grids as part of a translocation experiment conducted in 2018 and 2019 [42], giving us data from an overall total of 46 raccoons for use in our analyses. For these supplemental animals, we only used data from individuals prior to translocation so that movement behaviors were not affected by experimental manipulation. All animal handling practices conformed to the American Society of Mammologists guidelines [41]. All trapping and handling were conducted in accordance with the University of Georgia Animal Care and Use Guidelines under Animal Care and Use Protocol A2018 06-024-A12. Field activities were approved by the Site Use Program of the Savannah River Site under Site Use Permit SU-20-42-R.

### Analysis

We filtered out GPS points that did not meet the criteria of having 4 satellites at the time of location. We also excluded months from each animal’s location data that had less than 30 recorded GPS points, and partial months of data from the start and end of each individual’s monitoring period. From the remaining points, we calculated the monthly home ranges and core use areas of every individual by constructing 95% and 60% utilization distributions (UDs), respectively, using dynamic Brownian bridge models with the package ‘move’ [43] in Program R version 4.2.1 [44]. We constructed two sets of linear mixed effects models using the package lme4, with the two log-transformed UD sizes as the two response variables. We included individual as a random effect and fixed effects included habitat, sex, and season. We defined seasons that were biologically meaningful for raccoons in the Southeastern US: breeding = 1 Feb–31 May; young-rearing/summer, hereafter summer = 1 Jun–30 Sept; and fall/winter, hereafter fall = 1 Oct–31 Jan [8, 24]. We also included all possible interactions between the three fixed effects in our models.

To evaluate models, we ranked null and all possible model combinations using sample size corrected AIC_c_, selecting the lowest AIC_c_ as the best performing model and making inferences from this top model [45]. We also assessed the relative support for the top model by calculating the Akaike weights (*w*_*i*_) and comparing it to other competitive models, defined as those within two AIC_c_ units. We used the package ‘emmeans’ to compute pairwise comparisons between variables in the top model using α = 0.05 to determine statistical significance [46].

## Results

**Fig 2:**
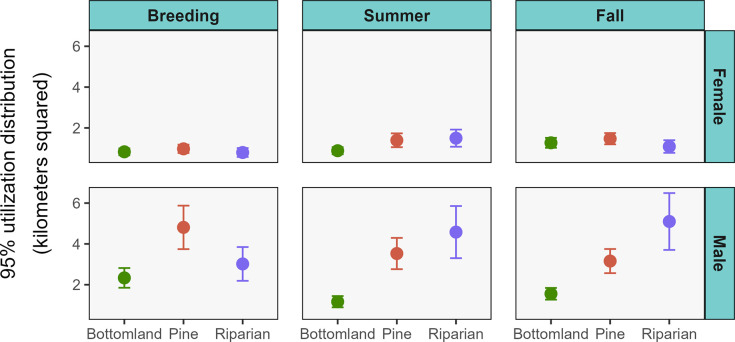
Estimated 95% utilization distributions of raccoons monitored across three habitats at the Savannah River Site, South Carolina, USA (2018–2019; 2021–2022). Seasons were breeding (1 Feb–31 May), summer (1 Jun–30 Sept), and fall (1 Oct–31 Jan).

**Fig 3:**
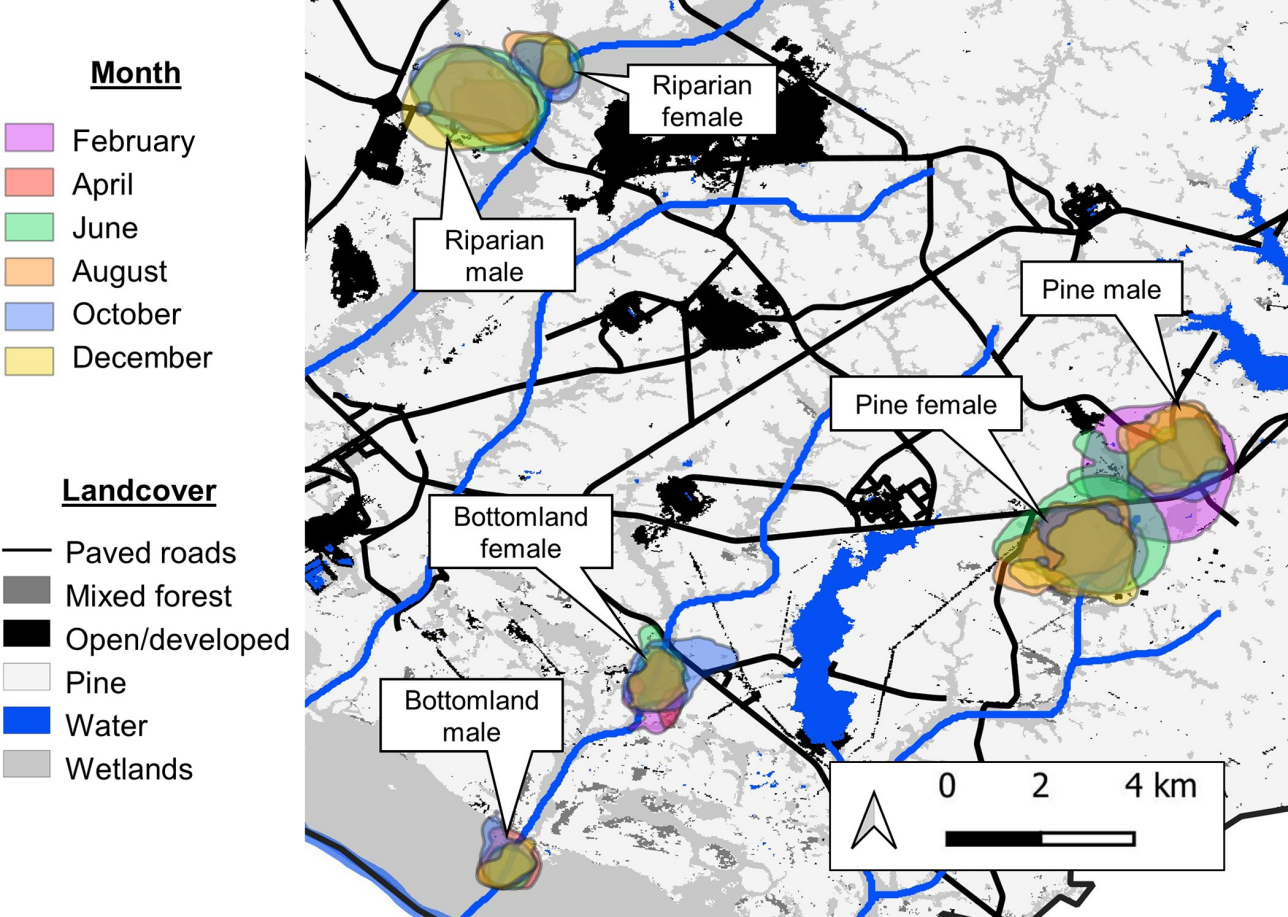
Monthly 95% utilization distribution of 3 female and 3 male raccoons in riparian, bottomland hardwood, and upland pine habitats on the Savannah River Site, Aiken, South Carolina, USA (2021–2022).

We obtained 264 monthly home ranges from 46 raccoons. Our 95% UD sizes were influenced by the interaction between sex, habitat, and season, with no other competitive models (S1 Table). Across these treatment combinations, mean monthly 95% UD sizes of raccoons ranged from 1.05 ± 0.48 km^2^ (breeding season bottomland females) to 5.69 ± 3.37 km^2^ (fall riparian males) (Table 1, Figs 2 and 3). Home ranges did not differ between pine and riparian for either sex in any season (S2 Table). Home ranges of males in autumn were about 2 and 3 times larger in pine and riparian habitats, respectively, compared to bottomlands. During summer, the differences were larger with male home ranges about 3 and 4 times greater in pine and riparian than in bottomland, respectively. During the breeding season, home ranges of males were twice as large in pine compared to bottomland habitat, but there was no difference in home range sizes between pine and riparian or bottomland and riparian. Home ranges of females were not different between habitats in any season. Males maintained larger home ranges than females throughout all seasons in pine and riparian habitats. These differences ranged from 2 times larger for males in pine during fall to 5 times larger in pine during breeding. Home ranges of males in bottomlands were 2.8 times larger than females during the breeding season, but not different during the other seasons.

**Table 1:** 95% and 60% utilization distribution (UD) sizes in km^2^ with standard deviation across habitats, sexes, and seasons of raccoons monitored on the Savannah River Site, Aiken, South Carolina, USA (2018–2019; 2021–2022). Sample sizes are presented as number of individuals monitored and total months of data provided by those individuals.

Habitat	Sex	Season	Sample size		95% UD	95% SD	60% UD	60% SD
			Individuals	Months				
Bottomland hardwood	Female	Breeding	5	18	1.05	0.48	0.25	0.15
		Summer	8	17	1.10	0.38	0.29	0.12
		Fall	9	21	1.67	1.08	0.40	0.24
	Male	Breeding	6	11	2.29	1.32	0.55	0.34
		Summer	4	7	1.13	1.24	0.29	0.27
		Fall	9	19	2.24	2.07	0.51	0.45
	Female		9	56	1.30	0.79	0.32	0.19
	Male		11	37	2.05	1.76	0.48	0.39
	**Overall**		**20**	**93**	**1.60**	**1.31**	**0.38**	**0.30**
Upland pine	Female	Breeding	5	15	1.85	1.80	0.48	0.51
		Summer	2	8	2.92	2.22	0.74	0.47
		Fall	8	27	1.97	1.31	0.45	0.30
	Male	Breeding	4	12	5.22	2.14	1.31	0.43
		Summer	5	12	3.70	0.86	0.96	0.29
		Fall	8	24	3.44	1.49	0.83	0.35
	Female		9	50	2.09	1.64	0.50	0.41
	Male		9	48	3.95	1.70	0.98	0.40
	**Overall**		**18**	**98**	**3.00**	**1.91**	**0.74**	**0.47**
Riparian forest	Female	Breeding	4	13	1.14	0.93	0.29	0.26
		Summer	3	12	2.03	0.73	0.46	0.18
		Fall	3	10	1.57	1.14	0.34	0.30
	Male	Breeding	4	13	3.20	1.45	0.85	0.46
		Summer	3	12	5.11	2.50	1.59	1.02
		Fall	4	13	5.69	3.37	1.30	0.75
	Female		4	35	1.57	0.98	0.36	0.25
	Male		4	38	4.65	2.72	1.24	0.81
	**Overall**		**8**	**73**	**3.17**	**2.58**	**0.82**	**0.75**

Comparing seasons within habitats, riparian males had average home ranges about 1.5 times greater in fall and summer compared to breeding. Riparian females had home ranges about twice as large in summer compared to breeding, but no differences between other seasons. In pine, males had home ranges in breeding season a third larger than in fall, but no differences between other seasons. For females in pine, the only difference was a 50% decrease in home range sizes during breeding compared fall. The only seasonal difference for bottomland males was a 50% increase in home range size in breeding compared to summer. Home ranges were 1.5 times larger for bottomland females during fall compared to breeding or summer.

**Fig 4:**
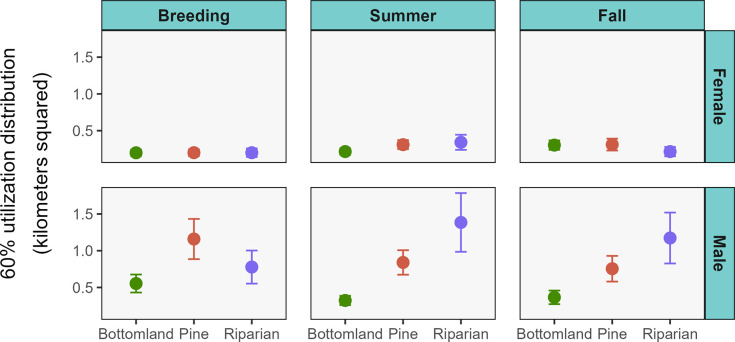
Estimated 60% utilization distributions of raccoons monitored across three habitats at the Savannah River Site, South Carolina, USA (2018–2019; 2021–2022). Seasons were breeding (1 Feb–31 May), summer (1 Jun–30 Sept), and fall (1 Oct–31 Jan).

We found the same general patterns when examining the core areas, with 60% UD sizes influenced by the interaction between sex, habitat, and season and no other competitive models (S3 Table). Mean monthly 60% UD sizes of raccoons ranged from 0.25 ± 0.15 km^2^ (breeding season bottomland females) to 1.59 ± 1.02 km^2^ (summer riparian males) (Table 1 and Fig 4). Core areas did not differ between pine and riparian for either sex in any season (S4 Table). Similar to the home range analysis, core area sizes of males were larger in both pine and riparian compared to bottomland. However, the increase in home range size of males in pine compared to bottomland during the breeding season documented for 95% UD home ranges was not present for core areas. Core areas of females did not differ in size between habitats in any season. Core area sizes were larger for males than females in every season and habitat but there was no difference in core area sizes between the sexes in fall or summer in the bottomlands. Results of comparisons of seasonal core areas within habitats were the same for riparian male and females as in the 95% UD home range analysis. The only seasonal difference in core areas for either sex in bottomland habitat was a 50% increase in core area size of females in fall compared to the breeding season.

## Discussion

Raccoon home range and core area sizes in rural habitats of the southeastern United States were influenced by interactions between season, habitat, and sex. Contrary to our predictions, home ranges and core areas were not smaller overall in bottomland hardwoods or riparian compared to upland pine, and habitat-specific differences were sex dependent. Space use area of males was smaller in bottomlands compared to upland pine as predicted, but there was no difference between upland pine and riparian habitats. For females, however, the same relationship was not observed as female home ranges and core areas were equal across habitats.

The home range and core area sizes of raccoons in bottomland habitat were consistent with other studies in non-urban habitats [7, 8, 13, 24, 47]. The home ranges of males in upland pine and riparian habitat, however, are among the largest home ranges recorded for raccoons in the United States [9]. Correspondingly, raccoon densities in these habitats are among the smallest reported [35], consistent with pine being one of the least preferred habitats by raccoons [48, 49].

Differential resource availability likely leads to sex-specific patterns of space use across habitats. Bottomland hardwoods are presumed to be resource abundant due to the presence of large diameter trees for denning and free water [8, 23, 24]. This assumption at our site is supported by the similar home range and core areas sizes between males and females in bottomlands, whereas male home ranges and core areas were consistently several times larger than females in the other habitats. Male raccoons are larger than females which leads to larger home ranges to fulfill resource requirements [7, 24, 26], but the concentration of resources in bottomland hardwoods requires males to move less widely to meet resource requirements than in other habitats, resulting in more similar space use between the sexes. Likewise, there was no sexual dimorphism in raccoon home range sizes in Illinois, where home range sizes between males and females became increasingly similar as habitats became more resource abundant [50]. Although male raccoons generally have larger home ranges than females, this pattern may be mediated somewhat by resource abundance and distribution.

The large home range sizes of male raccoons in riparian habitat contradict our assumption that this habitat was equivalent to bottomland hardwood in terms of resource availability. This may result from the patchier configuration of riparian habitat within pine compared to the large contiguous block that characterizes bottomland hardwood habitat on our site. Riparian habitat does not seem to exist in large enough patches for males to meet resource requirements solely within that habitat type, requiring them to incorporate adjacent pine habitat and resulting in large home ranges [51]. Similarly, raccoons in agricultural habitat in Indiana had large home ranges when they inhabited small forest patches because they needed to use multiple patches to meet resource requirements, whereas home ranges were smaller in large blocks of contiguous forest [15]. Riparian habitat on our site appears sufficient to fulfill the smaller resource requirements of females as female home ranges and core areas were similar in size across the habitats examined.

Raccoon density estimates in these habitats support our conclusions because raccoon densities are largely influenced by resource availability [13, 52]. At the SRS, bottomland hardwoods have higher raccoon densities (mean density = 5.44 ± 0.37 animals/km^2^) than upland pine (2.14 ± 0.23 animals/km^2^) or riparian (2.62 ± 0.32 animals/km^2^) habitat, whereas densities between the latter two do not differ [35]. This pattern is consistent with bottomland hardwoods having higher resource availability resulting in smaller home ranges and higher population densities. In these same habitats on the SRS, however, opossum densities are similar between bottomland and riparian, but comparatively lower than in pine [53]. Opossums have smaller home ranges than raccoons in similar habitats due to smaller body mass and decreased resource requirements [54]. Across the spectrum of resource requirements, there is an apparent threshold between those of opossums/female raccoons and male raccoons beyond which riparian habitat on the SRS is insufficient to fulfill.

We did not find clear support for our predictions regarding seasonal changes in space use, which were not consistent across habitats or sexes. In upland pine, males had the largest home ranges in the breeding season as predicted, whereas riparian males exhibited their smallest home ranges during the breeding season. This pattern may relate to den tree availability in the riparian habitat that was not similarly available in the pine habitat, constricting males to these well-defined areas where females were located [55]. Riparian females showed patterns opposite of our predictions, having their largest home ranges in summer and smallest during breeding. These variable seasonal patterns are in accordance with previous findings as there does not seem to be a consistent relationship between season and home range size for raccoons across study populations [8, 24–26]. In Texas, there was no overall change in median raccoon home range size for either sex seasonally, although there were seasonal changes for certain individuals [26]. Our data supports this conclusion as within sex and habitat levels some individuals demonstrated relatively static home range and core area sizes throughout the year whereas space use of others changed markedly. Thus, individual variability may play a sizeable role in seasonal changes in home range sizes, limiting the ability to make seasonal inferences at the population level.

Although disease transmission risk is higher at elevated raccoon densities [56], our results indicate that sparsely populated upland pine and riparian habitats are still a concern for disease transmission due to extensive movements of males. Individuals with large home ranges are more likely to become infected and spread diseases (e.g., RABV, canine distemper) due to contact with many conspecifics over a large spatial scale [16, 17, 22]. Home ranges with standard deviations of several square kilometers for males in these habitats suggests considerable variability in home range sizes, which further increases the risk of disease spread [17]. As a result, male raccoons in pine and riparian areas of the southeastern US may contribute disproportionately to landscape level disease spread. Population immunity of RABV is impacted by raccoon contact rates across the rural-urban gradient [57]. Managed pine forests like those on the SRS, which currently comprise 13% of the southeastern United States land area, are expected to increase in coming decades [58], potentially causing greater contact between urban and rural raccoons. Continued RABV monitoring and management in pine and riparian habitats across the southeastern United Stated will, therefore, be important to landscape level RABV reduction.

## Supporting information

S1 Table Generalized linear model comparisons for 95% utilization distribution area of raccoons at the Savannah River Site, Aiken, South Carolina, USA (2018–2019; 2021–2022). Levels of habitat are bottomland hardwood, upland pine, and riparian. Seasons were breeding (1 Feb–31 May), summer (1 Jun–30 Sept), and fall (1 Oct–31 Jan). Model output includes sample size corrected Akaike’s information criterion (AIC_c_), Akaike weights (*w*_*i*_), log likelihood (*LL*), number of parameters (*K)*, and difference in AIC_c_ between each model and top model (ΔAIC_c_). Plus sign (+) in columns indicates the parameter was included in the model.
(PDF)

S2 Table 95% utilization distribution size comparisons between habitats, seasons, and sexes of raccoons monitored on the Savannah River Site, Aiken, South Carolina, USA (2018–2019; 2021–2022). Bold values along the center diagonal are the model estimated 95% UD sizes (in km^2^) for that group. Values below the diagonal are the p-values for group comparisons (red = significant at *p* < 0.05) and above the diagonal is the corresponding estimated difference (group in the column divided by group in the row; blue = significant at *p* < 0.05). Gray boxes are comparisons that are not relevant to the study (e.g., less than 2 shared treatment levels).
(PDF)

S3 Table Generalized linear model comparisons for 60% utilization distribution area of raccoons at the Savannah River Site, Aiken, South Carolina, USA (2018–2019; 2021–2022). Levels of habitat are bottomland hardwood, upland pine, and riparian. Seasons were breeding (1 Feb–31 May), summer (1 Jun–30 Sept), and fall (1 Oct–31 Jan). Model output includes sample size corrected Akaike’s information criterion (AIC_c_), Akaike weights (*w*_*i*_), log likelihood (*LL*), number of parameters (*K)*, and difference in AIC_c_ between each model and top model (ΔAIC_c_). Plus sign (+) in columns indicates the parameter was included in the model.
(PDF)

S4 Table 60% utilization distribution size comparisons between habitats, seasons, and sexes of raccoons monitored on the Savannah River Site, Aiken South Carolina, USA (2018–2019; 2021–2022). Bold values along the center diagonal are the model estimated 60% UD sizes (in km^2^) for that group. Values below the diagonal are the p-values for group comparisons and above the diagonal is the corresponding estimated difference (group in the column divided by group in the row). Values in red indicate significant differences at p<0.05. Gray boxes are comparisons that are not relevant to the study (e.g., less than 2 shared treatment levels).
(PDF)

S1 File Telemetry data of raccoons on the Savannah River Site, Aiken, South Carolina, USA (2018–2019; 2021–2022). (XLSX)

## References

[1] Grace D, Gilbert J, Randolph T, Kang’ethe E. The multiple burdens of zoonotic disease and an ecohealth approach to their assessment. Trop Anim Health Prod. 2012;44(1):67–73. doi: 10.1007/s11250-012-0209-y 22886445

[2] Dougherty ER, Seidel DP, Carlson CJ, Spiegel O, Getz WM. Going through the motions: incorporating movement analyses into disease research. Ecol Lett. 2018;21(4):588–604. doi: 10.1111/ele.12917 29446237

[3] Abrahms B, Aikens EO, Armstrong JB, Deacy WW, Kauffman MJ, Merkle JA. Emerging perspectives on resource tracking and animal movement ecology. Trends Ecol Evol. 2021;36(4):308–20. doi: 10.1016/j.tree.2020.10.018 33229137

[4] Elmore SA, Chipman RB, Slate D, Huyvaert KP, VerCauteren KC, Gilbert AT. Management and modeling approaches for controlling raccoon rabies: The road to elimination. Plos Neglect Trop D. 2017;11(3):e0005249. doi: 10.1371/journal.pntd.0005249 28301480 PMC5354248

[5] Slate D, Rupprecht CE, Rooney JA, Donovan D, Lein DH, Chipman RB. Status of oral rabies vaccination in wild carnivores in the United States. Virus Res. 2005;111(1):68–76. doi: 10.1016/j.virusres.2005.03.012 15896404

[6] Slate D, Rupprecht C, Donovan D, Badcock J, Messier A, Chipman R, et al. Attaining raccoon rabies management goals: history and challenges. Developments in biologicals. 2008;131:439–48. 18634506

[7] Beasley JC, Devault TL, Rhodes OE Jr. Home‐range attributes of raccoons in a fragmented agricultural region of northern Indiana. J Wildl Manage. 2007;71(3):844–50.

[8] Byrne ME, Chamberlain MJ. Seasonal space use and habitat selection of adult raccoons (Procyon lotor) in a Louisiana bottomland hardwood forest. Am Midl Nat. 2011;166(2):426–34.

[9] Šálek M, Drahníková L, Tkadlec E. Changes in home range sizes and population densities of carnivore species along the natural to urban habitat gradient. Mamm Rev. 2015;45(1):1–14.

[10] O’Donnell K, del Barco-Trillo J. Changes in the home range sizes of terrestrial vertebrates in response to urban disturbance: a meta-analysis. Journal of Urban Ecology. 2020;6(1):juaa014.

[11] Beasley J, Dharmarajan G, Rhodes O Jr. Melding kin structure and demography to elucidate source and sink habitats in fragmented landscapes. Ecosphere. 2015;6(4):1–16.

[12] Adams ES. Approaches to the study of territory size and shape. Annu Rev Ecol Syst. 2001;32(1):277–303.

[13] Rosatte R, Ryckman M, Ing K, Proceviat S, Allan M, Bruce L, et al. Density, movements, and survival of raccoons in Ontario, Canada: implications for disease spread and management. J Mammal. 2010;91(1):122–35.

[14] Tardy O, Massé A, Pelletier F, Mainguy J, Fortin D. Density‐dependent functional responses in habitat selection by two hosts of the raccoon rabies virus variant. Ecosphere. 2014;5(10):1–16.

[15] Beasley J, Rhodes O. Influence of patch-and landscape-level attributes on the movement behavior of raccoons in agriculturally fragmented landscapes. Can J Zool. 2010;88(2):161–9.

[16] McClure KM, Bastille‐Rousseau G, Davis AJ, Stengel CA, Nelson KM, Chipman RB, et al. Accounting for animal movement improves vaccination strategies against wildlife disease in heterogeneous landscapes. Ecol Appl. 2022:e2568. doi: 10.1002/eap.2568 35138667 PMC9285612

[17] McClure KM, Gilbert AT, Chipman RB, Rees EE, Pepin KM. Variation in host home range size decreases rabies vaccination effectiveness by increasing the spatial spread of rabies virus. J Anim Ecol. 2020;89(6):1375–86. doi: 10.1111/1365-2656.13176 31957005 PMC7317853

[18] Rees EE, Pond BA, Tinline RR, Bélanger D. Modelling the effect of landscape heterogeneity on the efficacy of vaccination for wildlife infectious disease control. J Appl Ecol. 2013;50(4):881–91.

[19] Rees EE, Pond BA, Phillips JR, Murray D. Raccoon ecology database: a resource for population dynamics modelling and meta-analysis. Ecological Informatics. 2008;3(1):87–96.

[20] Beasley JC, Atwood TC, Byrne ME, Vercauteren KC, Johnson SR, Rhodes OE Jr. A behaviorally-explicit approach for delivering vaccine baits to mesopredators to control epizootics in fragmented landscapes. PLoS One. 2015;10(1):e0113206. doi: 10.1371/journal.pone.0113206 25587900 PMC4294636

[21] Tardy O, Lenglos C, Lai S, Berteaux D, Leighton PA. Rabies transmission in the Arctic: An agent-based model reveals the effects of broad-scale movement strategies on contact risk between Arctic foxes. Ecol Model. 2023;476:110207.

[22] Sauvé CC, Rees EE, Gilbert AT, Berentsen AR, Allibert A, Leighton PA. Modeling mongoose rabies in the caribbean: A model-guided fieldwork approach to identify research priorities. Viruses. 2021;13(2):323. doi: 10.3390/v13020323 33672496 PMC7923793

[23] Leberg PL, Kennedy ML. Demography and habitat relationships of raccoons in western Tennessee. Proceedings of the Annual Conference of Southeastern Association of Fish and Wildlife Agencies. 1988;42:272–82.

[24] Chamberlain MJ, Conner LM, Leopold BD, Hodges KM. Space use and multi-scale habitat selection of adult raccoons in central Mississippi. J Wildl Manage. 2003;67:334–40.

[25] Owen SF, Berl JL, Edwards JW. Raccoon spatial requirements and multi-scale habitat selection within an intensively managed central Appalachian forest. Am Midl Nat. 2015;174(1):87–95.

[26] Gehrt SD, Frttzell EK. Sexual differences in home ranges of raccoons. J Mammal. 1997;78(3):921–31.

[27] Chamberlain MJ, Conner LM, Leopold BD. Seasonal habitat selection by raccoons (Procyon lotor) in intensively managed pine forests of central Mississippi. Am Midl Nat. 2002;147(1):102–8.

[28] Beasley JC, Beatty WS, Atwood TC, Johnson SR, Rhodes OE Jr. A comparison of methods for estimating raccoon abundance: implications for disease vaccination programs. J Wildl Manage. 2012;76(6):1290–7.

[29] Samuel MD, Pierce D, Garton EO. Identifying areas of concentrated use within the home range. The Journal of Animal Ecology. 1985:711–9.

[30] White DL, Gaines KF. The Savannah River Site: site description, land use, and management history. Studies in Avian Biology. 2000;21:8–17.

[31] Yang L, Jin S, Danielson P, Homer C, Gass L, Bender SM, et al. A new generation of the United States National Land Cover Database: Requirements, research priorities, design, and implementation strategies. ISPRS Journal of Photogrammetry and Remote Sensing. 2018;146:108–23.

[32] Stutter M, Baggaley N, Wang C. The utility of spatial data to delineate river riparian functions and management zones: A review. Sci Total Environ. 2021;757:143982. doi: 10.1016/j.scitotenv.2020.143982 33310572

[33] Thomas IV JC, Oladeinde A, Kieran TJ, Finger JW Jr, Bayona‐Vásquez NJ, Cartee JC, et al. Co‐occurrence of antibiotic, biocide, and heavy metal resistance genes in bacteria from metal and radionuclide contaminated soils at the Savannah River Site. Microbial Biotechnology. 2020;13(4):1179–200. doi: 10.1111/1751-7915.13578 32363769 PMC7264878

[34] Layman SR. Life history of the Savannah darter, Etheostoma fricksium, in the Savannah River drainage, South Carolina. Copeia. 1993:959–68.

[35] Hill J, Helton J, Bernasconi D, Dixon WC, Hamilton M, Chipman R, et al. Raccoon densities across four rural habitats in the Southeastern United States. Journal of Wildilfe Management. 2023; 87(8): e22480.

[36] Webster SC, Beasley JC. Influence of lure choice and survey duration on scent stations for carnivore surveys. Wildl Soc Bull. 2019;43(4):661–8.

[37] Smyser TJ, Beasley JC, Olson ZH, Rhodes OE Jr. Use of rhodamine B to reveal patterns of interspecific competition and bait acceptance in raccoons. J Wildl Manage. 2010;74(6):1405–16.

[38] Gehrt SD, Hungerford LL, Hatten S. Drug effects on recaptures of raccoons. Wildl Soc Bull. 2001;29:833–7.

[39] Beasley JC, Rhodes OE Jr. Relationship between raccoon abundance and crop damage. Human-Wildlife Conflicts. 2008;2(2):248–59.

[40] Wilson DE, Cole RF, Nichols JD, Foster MS. Measuring and monitoring biological diversity: standard methods for mammals. Washington DC: Smithsonian Books; 1996. 409 p.

[41] Sikes RS, Care A, Mammalogists UCotASo. 2016 Guidelines of the American Society of Mammalogists for the use of wild mammals in research and education. J Mammal. 2016;97(3):663–88. 29692469 10.1093/jmammal/gyw078 PMC5909806

[42] Hill JE, Helton J, Gilbert A, Chipman R, Beasley J, Dharmarajan G, et al. Spatial ecology of translocated raccoons. Sci Rep. 2023;13(1):10447. doi: 10.1038/s41598-023-37323-6 37369730 PMC10300129

[43] Kranstauber B, Smolla M, Scharf AK, Kranstauber MB. Package ‘move’. 2020.

[44] R Core Team. R: A language and environment for statistical computing. R Foundation for Statistical Computing. Vienna, Austria 2022.

[45] Burnham KP, Anderson DR. Model selection and multimodel inference: a practical information-theoretical approach. New York: Springer; 2002. 488 p.

[46] Lenth R, Singmann H, Love J, Buerkner P, Herve M. Emmeans: Estimated marginal means, aka least-squares means. R package version. 2018;1(1):3.

[47] Gross J, Elvinger F, Hungerford LL, Gehrt SD. Raccoon use of the urban matrix in the Baltimore Metropolitan Area, Maryland. Urban Ecosyst. 2012;15(3):667–82.

[48] Klimas C, Martin C, Teaford J. Impacts of flooding regime modification on wildlife habitats of bottomland hardwood forests in the lower Mississippi Valley. Vicksburg, MS: US Army Engineer Division; 1981. Contract No.: Technical report EL-81-13.

[49] Slate D, Saidy BD, Simmons A, Nelson KM, Davis A, Algeo TP, et al. Rabies management implications based on raccoon population density indexes. J Wildl Manage. 2020;84(5):877–90.

[50] Bozek CK, Prange S, Gehrt SD. The influence of anthropogenic resources on multi-scale habitat selection by raccoons. Urban Ecosyst. 2007;10(4):413–25.

[51] Clontz LM, Pepin KM, VerCauteren KC, Beasley JC. Behavioral state resource selection in invasive wild pigs in the Southeastern United States. Sci Rep. 2021;11(1):6924. doi: 10.1038/s41598-021-86363-3 33767284 PMC7994638

[52] Beasley JC, Olson ZH, Dharmarajan G, Eagan TS, Rhodes OE. Spatio-temporal variation in the demographic attributes of a generalist mesopredator. Landscape Ecol. 2011;26(7):937–50.

[53] Bernasconi DA, Dixon WC, Hamilton MT, Helton JL, Chipman RB, Gilbert AT, et al. Influence of landscape attributes on Virginia opossum density. J Wildl Manage. 2022;86(7):e22280.

[54] Ginger SM, Hellgren EC, Kasparian MA, Levesque LP, Engle DM, Leslie DM. Niche shift by Virginia opossum following reduction of a putative competitor, the raccoon. J Mammal. 2003;84(4):1279–91.

[55] Beasley JC, Rhodes OE. Are raccoons limited by the availability of breeding resources? Evidence of variability in fecundity at fine spatial scales. J Wildl Manage. 2012;76(8):1718–24.

[56] Houle M, Fortin D, Mainguy J, Canac-Marquis P. Landscape composition and structure influence the abundance of mesopredators: implications for the control of the raccoon (Procyon lotor) variant of rabies. Can J Zool. 2011;89(11):1107–16.

[57] Davis AJ, Nelson KM, Kirby JD, Wallace R, Ma X, Pepin KM, et al. Rabies surveillance identifies potential risk corridors and enables management evaluation. Viruses. 2019;11(11):1006. doi: 10.3390/v11111006 31683632 PMC6893774

[58] Zhang D, Polyakov M. The geographical distribution of plantation forests and land resources potentially available for pine plantations in the US South. Biomass Bioenergy. 2010;34(12):1643–54.

