# Supplementary material for: Raccoon spatial ecology in the rural southeastern United States
Source: PLoS One. 2023 Nov 9;18(11):e0293133. doi: 10.1371/journal.pone.0293133 (PMC10635488; doi:10.1371/journal.pone.0293133)
Supplement: S1 Table — Levels of habitat are bottomland hardwood, upland pine, and riparian. Seasons were breeding (1 Feb–31 May), summer (1 Jun–30 Sept), and fall (1 Oct–31 Jan). Model output includes sample size corrected Akaike’s information criterion (AICc), Akaike weights (wi), log likelihood (LL), number of parameters (K), and difference in AICc between each model and top model (ΔAICc). Plus sign (+) in columns indicates the parameter was included in the model. (PDF) [file pone.0293133.s001.pdf]

**S1 Table. Generalized linear model comparisons for 95% utilization distribution area of raccoons at the Savannah River Site, South Carolina, USA (2018-2019; 2021-2022).** Levels of habitat are bottomland hardwood, upland pine, and riparian. Seasons were breeding (1 Feb–31 May), summer (1 Jun–30 Sept), and fall (1 Oct–31 Jan). Model output includes sample size corrected Akaike's information criterion ($AIC_c$), Akaike weights ($w_i$), log likelihood (LL), number of parameters (*K*), and difference in $AIC_c$ between each model and top model ($\Delta AIC_c$). Plus sign (+) in columns indicates the parameter was included in the model.

| **Habitat** | **Season** | **Sex** | **Habitat: season** | **Habitat: sex** | **Season: sex** | **Habitat: season: sex** | ***K*** | ***LL*** | **$AIC_c$** | **$\Delta AIC_c$** | **$w_i$** |
|---|---|---|---|---|---|---|---|---|---|---|---|
| + | + | + | + | + | + | + | 20 | -172.92 | 389.30 | 0.00 | 0.85 |
| + | + | + | + | + | + | | 16 | -179.90 | 394.00 | 4.70 | 0.08 |
| + | + | + | + | | + | | 14 | -182.30 | 394.28 | 4.97 | 0.07 |
| + | + | + | + | + | | | 14 | -189.23 | 408.15 | 18.85 | 0.00 |
| + | + | + | | | + | | 10 | -193.76 | 408.39 | 19.08 | 0.00 |
| + | + | + | + | | | | 12 | -191.61 | 408.47 | 19.16 | 0.00 |
| + | + | + | | + | + | | 12 | -191.75 | 408.74 | 19.44 | 0.00 |
| | + | + | | | + | | 8 | -198.13 | 412.83 | 23.52 | 0.00 |
| + | + | + | | | | | 8 | -200.29 | 417.15 | 27.84 | 0.00 |
| + | + | + | | + | | | 10 | -198.25 | 417.36 | 28.06 | 0.00 |
| | + | + | | | | | 6 | -204.39 | 421.10 | 31.80 | 0.00 |
| + | | + | | + | | | 8 | -202.73 | 422.02 | 32.72 | 0.00 |
| + | | + | | | | | 6 | -204.88 | 422.08 | 32.77 | 0.00 |
| | | + | | | | | 4 | -209.15 | 426.45 | 37.15 | 0.00 |
| + | + | | + | | | | 11 | -202.08 | 427.21 | 37.90 | 0.00 |
| + | + | | | | | | 7 | -210.69 | 435.82 | 46.51 | 0.00 |
| | + | | | | | | 5 | -213.02 | 436.27 | 46.97 | 0.00 |
| + | | | | | | | 5 | -215.56 | 441.35 | 52.05 | 0.00 |
| | | | | | | | 3 | -217.98 | 442.05 | 52.75 | 0.00 |
