# Supplementary material for: Raccoon spatial ecology in the rural southeastern United States
Source: PLoS One. 2023 Nov 9;18(11):e0293133. doi: 10.1371/journal.pone.0293133 (PMC10635488; doi:10.1371/journal.pone.0293133)
Supplement: S2 Table — Bold values along the center diagonal are the model estimated 95% UD sizes (in km2) for that group. Values below the diagonal are the p-values for group comparisons (red = significant at p < 0.05) and above the diagonal is the corresponding estimated difference (group in the column divided by group in the row; blue = significant at p < 0.05). Gray boxes are comparisons that are not relevant to the study (e.g., less than 2 shared treatment levels). (PDF) [file pone.0293133.s002.pdf]

S2 Table. 95% utilization distribution size comparisons between habitats, seasons, and sexes of raccoons monitored on the Savannah River Site, Aiken SC, USA (2018-2019; 2021-2022). Bold values along the center diagonal are the model estimated 95% UD sizes (in km^2) for that group. Values below the diagonal are the p-values for group comparisons (red = significant at $p < 0.05$) and above the diagonal is the corresponding estimated difference (group in the column divided by group in the row; blue = significant at $p < 0.05$). Gray boxes are comparisons that are not relevant to the study (e.g., less than 2 shared treatment levels).

			Bottomland						Upland pine						Riparian					
			Breeding		Summer		Fall		Breeding		Summer		Fall		Breeding		Summer		Fall	
			F	M	F	M	F	M	F	M	F	M	F	M	F	M	F	M	F	M
Bottomland	Breeding	F	**0.83**	2.81	1.06		1.53		1.18						0.96					
		M	0.01	**2.33**		0.50		0.67		2.06						1.30				
	Summer	F	0.90		**0.88**	1.33	1.44				1.59						1.70			
		M		0.01	0.37	**1.17**		1.33				3.02						3.91		
	Fall	F	0.01		0.02		**1.27**	1.23					1.16						0.86	
		M		0.06		0.37	0.45	**1.56**						2.03						3.27
Upland pine	Breeding	F	0.85						**0.98**	4.91	1.43		1.50		0.82					
		M		0.05					0.01	**4.81**		0.73		0.66		0.63				
	Summer	F			0.32				0.13		**1.40**	2.52	1.05				1.07			
		M				0.01				0.15	0.01	**3.53**		0.90				1.30		
	Fall	F					0.85		0.02		0.78		**1.47**	2.15					0.74	
		M						0.02		0.04		0.20	0.01	**3.16**						1.61
Riparian	Breeding	F	0.99						0.83						**0.80**	3.78	1.88		1.36	
		M		0.74						0.38					0.01	**3.02**		1.52		1.69
	Summer	F			0.28						0.98				0.01		**1.50**	3.05	0.73	
		M				0.01						0.74				0.03	0.01	**4.58**		1.11
	Fall	F					0.89						0.65		0.17		0.20		**1.09**	4.20
		M						0.01						0.32		0.01		0.80	0.01	**5.10**
