# Supplementary material for: Raccoon spatial ecology in the rural southeastern United States
Source: PLoS One. 2023 Nov 9;18(11):e0293133. doi: 10.1371/journal.pone.0293133 (PMC10635488; doi:10.1371/journal.pone.0293133)
Supplement: S4 Table — Bold values along the center diagonal are the model estimated 60% UD sizes (in km2) for that group. Values below the diagonal are the p-values for group comparisons and above the diagonal is the corresponding estimated difference (group in the column divided by group in the row). Values in red indicate significant differences at p<0.05. Gray boxes are comparisons that are not relevant to the study (e.g., less than 2 shared treatment levels). (PDF) [file pone.0293133.s004.pdf]

S4 Table. 60% utilization distribution size comparisons between habitats, seasons, and sexes of raccoons monitored on the Savannah River Site, Aiken SC, USA (2018-2019; 2021-2022). Bold values along the center diagonal are the model estimated 60% UD sizes (in km^2) for that group. Values below the diagonal are the p-values for group comparisons and above the diagonal is the corresponding estimated difference (group in the column divided by group in the row). Values in red indicate significant differences at $p<0.05$. Gray boxes are comparisons that are not relevant to the study (e.g., less than 2 shared treatment levels).

			Bottomland						Upland pine						Riparian					
			Breeding		Summer		Fall		Breeding		Summer		Fall		Breeding		Summer		Fall	
			F	M	F	M	F	M	F	M	F	M	F	M	F	M	F	M	F	M
Bottomland	Breeding	F	**0.20**	**2.75**	1.10		**1.50**		1.00						1.00					
		M	**0.01**	**0.55**		0.58		**0.67**		2.11						1.42				
	Summer	F	0.87		**0.22**	1.45	**1.36**				1.41						1.55			
		M		0.08	0.22	**0.32**		0.63				**0.97**						**0.69**		
	Fall	F	**0.01**		**0.05**		**0.30**	1.23					1.03						0.73	
		M		**0.01**		0.86	0.51	**0.37**						**2.03**						**3.16**
Upland pine	Breeding	F	1.00						**0.20**	**5.80**	1.55		**1.55**		1.00					
		M		0.06					**0.01**	**1.16**		0.72		**0.65**		0.67				
	Summer	F			0.53				0.08		**0.31**	**2.71**	1.00				1.10			
		M				**0.02**				0.18	**0.01**	**0.84**		0.89				1.64		
	Fall	F					1.00		**0.02**		1.00		**0.31**	**2.42**					0.71	
		M						**0.03**		**0.05**		0.82	**0.01**	**0.75**						1.56
Riparian	Breeding	F	1.00						1.00						**0.20**	**3.90**	**1.70**		1.10	
		M		0.62						0.54					**0.01**	**0.78**		**1.77**		**1.50**
	Summer	F			0.42						1.00				**0.01**		**0.34**	**4.06**	0.65	
		M				**0.01**						0.38				**0.01**	**0.01**	**1.38**		0.85
	Fall	F					0.61						0.57		0.92		0.06		**0.22**	**6.27**
		M						**0.01**						0.42		**0.048**		0.63	**0.01**	**1.17**
